# Congenital diaphragmatic hernia subtypes: Comparing birth prevalence, occurrence by maternal age, and mortality in a national birth cohort

**DOI:** 10.1111/ppe.12939

**Published:** 2022-11-28

**Authors:** Maria Peppa, Bianca L. De Stavola, Stavros Loukogeorgakis, Ania Zylbersztejn, Ruth Gilbert, Paolo De Coppi

**Affiliations:** ^1^ Population, Policy and Practice Research and Teaching Department UCL Great Ormond Street Institute of Child Health London UK; ^2^ Stem Cell and Regenerative Medicine UCL Great Ormond Street Institute of Child Health London UK; ^3^ Specialist Neonatal and Paediatric Surgery Unit Great Ormond Street Hospital London UK

**Keywords:** congenital abnormalities, epidemiology, mortality, routinely collected health data

## Abstract

**Background:**

Population‐based administrative data have rarely been used to compare the birth prevalence, risk factors for occurrence, and mortality of congenital diaphragmatic hernia (CDH) subtypes.

**Objectives:**

We used a national birth cohort to identify CDH subtypes and compared their birth prevalence, relationship with maternal age after accounting for sociodemographic factors, and 1‐year mortality rates.

**Methods:**

Linked hospital admission and death records were used to identify isolated and complex CDH cases (involving additional anomalies) among singleton livebirths in England between 2002 and 2018. The prevalence of each CDH subtype per 10,000 livebirths was estimated overall and by infant, birth and maternal characteristics. The relationship between maternal age and each subtype relative to no CDH was examined using multivariable log‐binomial regression to estimate risk ratios (RRs). One‐year mortality rates were examined using Kaplan–Meier curves and the hazard ratio (HR) of complex versus isolated CDH was calculated using Cox regression.

**Results:**

Among 9.5 million livebirths, we identified 1285 with isolated CDH and 1150 with complex CDH. The overall prevalence of isolated and complex CDH was 1.4 (95% confidence interval [CI] 1.3, 1.4) and 1.2 (95% CI 1.1, 1.3) per 10,000 livebirths, respectively. Only complex CDH was associated with maternal age. Compared with maternal age 25–34 years, complex CDH risk was elevated for maternal age < 20 years (RR 1.31, 95% CI 1.00, 1.72). Risk was highest for maternal age ≥ 40 years (RR 1.61, 95% CI 1.21, 2.15) although accounting for chromosomal anomalies attenuated the risk (RR 1.39, 95% CI 1.00, 1.92). The 1‐year mortality rate for complex CDH (33.1%, 95% CI 30.5, 35.9) was slightly higher than for isolated CDH (29.7%, 95% CI 27.3, 32.3) (HR 1.10, 95% CI 0.96, 1.27).

**Conclusions:**

Mechanisms of occurrence differed between and within CDH subtypes and 1‐year mortality of complex CDH was slightly higher than for isolated CDH.

**Synopsis**

**Study Question**

How do congenital diaphragmatic hernia (CDH) subtypes compare in their birth prevalence, occurrence across different maternal age groups and mortality?

**What is Already Known?**

Few epidemiological studies have described the birth prevalence of subtypes of CDH or examined potential risk factors for their occurrence. Current mortality estimates for subtypes are limited by the use of registry data, which may be subject to ascertainment bias.

**What this Study Adds?**

We identified differences in the association of CHD subtypes with maternal age after accounting for sociodemographic factors which suggest different mechanisms of occurrence. We estimated 1‐year mortality rates for subtypes and examined mortality before and after surgery.

## 1 BACKGROUND

Congenital diaphragmatic hernia (CDH) is a rare condition with high mortality that affects 2.2–4.9 of every 10,000 births in England.^1^, ^2^ Treatment involves surgical repair of the diaphragm and, although there is no established timeframe, the consensus is for surgery to occur electively following cardiorespiratory stabilisation.^3^ CDH can occur in isolation or alongside other congenital anomalies (‘complex CDH’), but the epidemiology of these subtypes is not well understood.^1^, ^2^

Birth prevalence estimates for CDH subtypes have rarely been reported.^1^, ^2^ There is also a lack of evidence on their birth prevalence over time and by infant, birth and maternal characteristics.^1^, ^2^ Such evidence can help identify populations with greater burdens of disease, anticipate support needs and plan services.

Subtype aetiology is uncertain although genetic and maternal factors are likely involved.^4^, ^5^, ^6^, ^7^ Advanced maternal age increases the risk of chromosomal anomalies, which are estimated to affect 10% of CDH cases.^1^ Young maternal age is associated with factors related to social disadvantage, such as smoking or delayed antenatal care, that may increase the risk of certain congenital anomalies.^8^, ^9^, ^10^, ^11^, ^12^ Four US population‐based studies found no evidence of an association between maternal age and isolated CDH while no consensus emerged for complex CDH.^6^, ^13^, ^14^, ^15^ This has not been explored in the United Kingdom, and studies elsewhere may not be generalisable because maternal age is a proxy for reproductive, sociodemographic and behavioural risk factors with country‐specific distributions.^16^

Despite frequent assertions that CDH subtypes differ in mortality rates, the evidence base is sparse. A specialist registry study using data from UK paediatric surgical centres did not find evidence of an association between additional anomalies and 1‐year mortality of CDH but would miss cases born elsewhere that died before transfer.^17^ Conversely, older population‐based regional registry studies in the United Kingdom found greater 1‐year mortality among infants with additional anomalies, but study populations were small and case ascertainment by voluntary notifications could miss less severe cases.^18^, ^19^, ^20^

To address these limitations, we analysed all livebirths in England between 2002 and 2018 using linked hospital admission and death records which, unlike registry data, can explore the epidemiology of rare CDH subtypes at a national level while avoiding ascertainment bias. We described the birth prevalence of subtypes overall and by infant, birth and maternal characteristics. We assessed maternal age as a risk factor for each CDH subtype relative to no CDH after accounting for other sociodemographic factors. We also examined subtype mortality at 1 and 10 years of age, by birth period and by surgery status.

## 2 METHODS

### 2.1 Data sources

We used linked Hospital Episode Statistics (HES) and Office for National Statistics (ONS) death records. HES contains data from all state‐funded hospital admissions in England; 97% of births (livebirths and stillbirths) are captured and subsequent admissions of live‐born infants are linked.^21^ Linked maternal birth admissions can enhance information on maternal and birth characteristics.^22^ Diagnoses and procedures are encoded using the *International Classification of Diseases 10th Revision* (ICD‐10) and *Classification of Interventions and Procedures 4th Revision* (OPCS‐4), respectively. Linked death records include dates and all causes of death encoded using ICD‐10.

### 2.2 Study population

We carried out a retrospective study using a national cohort of singleton livebirths delivered between April 2002 and March 2018 to linked mothers resident in England.^22^ Follow‐up started at delivery and ended on the earliest of death, the first birthday (or tenth for the analysis of 10‐year mortality), or 31 March 2019.

### 2.3 Congenital diaphragmatic hernia definition

Infants were defined as having CDH if a diagnosis and repair were recorded in HES, or if CDH was recorded on the death certificate (Table S1). Infants with a sole diagnosis or repair in HES were considered cases if they had supportive evidence such as lung hypoplasia (Table S2). As CDH requires repair, those with a diagnosis but no repair during the first year of life also had their records reviewed by SL, a paediatric surgeon, to assess supportive evidence for inclusion. Infants were excluded if they were potentially misdiagnosed hiatus hernia cases or if CDH was incidental to exomphalos, gastroschisis or oesophageal malformations (Table S3).

Records of infants with CDH were searched for additional congenital anomaly diagnoses. The ICD‐10 codes used to define these were part of a wider code‐list developed by Hardelid et al (2013) to identify conditions requiring clinical follow‐up (Table S4).^23^ The Hardelid code list was developed iteratively with clinicians and underwent internal validation using HES.^23^, ^24^ Infants with CDH were classified into two groups. The ‘isolated’ group included infants without additional anomalies, or with related digestive or respiratory anomalies that occur as a sequence following developmental disruption of surrounding tissues. The ‘complex’ group included those with additional anomalies in other systems.

### 2.4 Other covariates

Data on birth year as well as infant, birth and maternal characteristics were available. Birth year was grouped into 4‐year periods. Infant characteristics included sex and ethnic group. Birth characteristics included gestational age at delivery and birthweight. Maternal characteristics included maternal age, maternal deprivation quintile (derived by allocating small‐area‐level Index of Multiple Deprivation scores to maternal residential post‐codes at delivery),^25^ and maternal region of residence, which may represent community and infrastructural influences on health not captured elsewhere.

### 2.5 Statistical analyses

Infant, birth and maternal characteristics of the study population were described by CDH status (none, any, isolated and complex). Overall birth prevalence per 10,000 livebirths was calculated for any, isolated and complex CDH. Birth prevalence of isolated and complex subtypes were reported by birth period, infant, birth and maternal characteristics.

Risk ratios (RRs) for isolated or complex CDH compared with none were estimated for maternal age groups using univariable and multivariable log‐binomial regression, with the latter accounting for birth period, infant ethnicity (a proxy for parental ethnicity which may be associated with maternal age and CDH occurrence), maternal deprivation and region (Box S1). The analyses restricted to infants for whom maternal age and these four potential confounders were recorded involved 82.4% (*n* = 7,807,310) of the original population (N = 9,473,825) and, thus, could be affected by selection bias. We, therefore, also performed multiple imputation (further details below); however, because missingness affected all variables similarly and is thought to relate to poor infant health^26^ which can only be partly captured by the available information, results may still be affected by selection bias.

Multicollinearity between ethnicity, deprivation and region was evaluated by monitoring the standard errors of their coefficients. To assess how much of the association between maternal age and complex CDH was accounted for by chromosomal anomalies we repeated the analysis excluding cases with chromosomal conditions.

The cumulative probability of death up to 1 year (1‐year mortality rate) for infants with isolated and complex CDH was estimated using Kaplan–Meier curves to account for varying follow‐up. The mortality hazard ratio (HR) and 95% confidence interval (95% CI) for complex versus isolated CDH was calculated using univariable Cox regression. One‐year mortality rates by birth period were examined by plotting separate Kaplan–Meier curves. Ten‐year mortality rates were also estimated.

The proportion of infants with each CDH subtype who died with and without surgical repair was calculated with 95% CIs. To assess the impact of surgery on mortality rates we fitted a proportional hazard model where surgery was treated as a time‐varying covariate.^27^ The proportional hazard assumption was evaluated graphically. Median days from birth to death and/or repair were estimated alongside interquartile ranges (IQRs).

### 2.6 Missing data

Missing data in the study population were observed for the following characteristics: infant sex (0.1%), infant ethnicity (15.9%), maternal age (2.1%), maternal deprivation (0.3%), gestational age (19.2%) and birth weight (14.6%). Analyses of maternal age and CDH occurrence were repeated using multiple imputations (under an assumption of missingness at random) with 10 imputed datasets generated using chained equations. The imputation model included all variables involved in the analysis of maternal age and CDH occurrence as well as gestational age and birth weight as missingness in birth characteristics was correlated with missingness of maternal age.

### 2.7 Sensitivity analyses

Missingness of infant, maternal and birth characteristics in HES may correlate with poorer infant health as clinical staff may have fewer opportunities to record these characteristics when infants have high‐care needs.^26^ Our complete record analysis of maternal age and risk of CDH subtypes included only individuals with complete infant and maternal characteristics. To assess whether these infants represented a healthier section of the CDH population, we compared them with the full CDH population. We repeated the comparison to the full CDH population with infants who had complete birth characteristics in addition to complete infant and maternal characteristics. The expectation was that these infants would be an even healthier section of the full CDH population. We conducted a sensitivity analysis for the risk of occurrence of CDH subtypes by maternal age in which we restricted to infants with complete infant, maternal and birth characteristics. Any changes in estimates would indicate the direction of bias in primary analyses restricted to infants with complete infant and maternal records.

### 2.8 Ethics approval

We have a data sharing agreement with National Health Service (NHS) Digital to use a de‐identified extract of HES linked to ONS death records; therefore, we did not require ethics approval to use English data sets.

## 3 RESULTS

### 3.1 Study population

**FIGURE 1:**
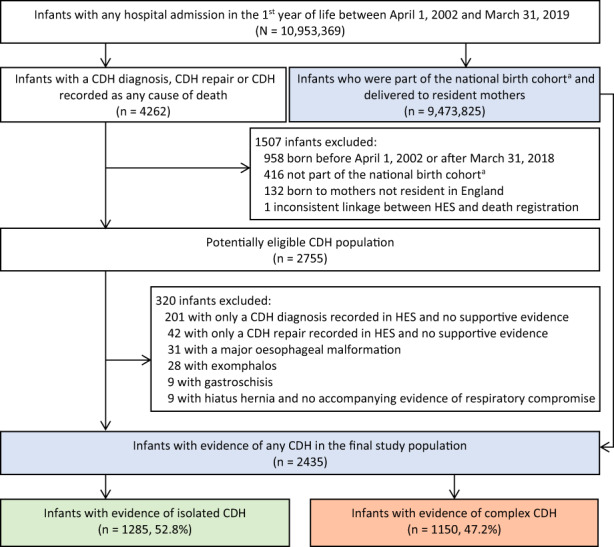
Derivation of the study population. ^a^Live‐born singletons delivered between April 1, 2002 and March 31, 2018. CDH, congenital diaphragmatic hernia; HES, Hospital Episode Statistics

We identified 2435 CDH cases among 9,473,825 live‐born singletons (Figure 1). CDH birth prevalence was 2.6 (95% CI 2.5, 2.7) per 10,000 livebirths. Of the identified CDH cases, 47.2% (*n* = 1150) were considered complex due to the presence of additional congenital anomalies outside of the respiratory and digestive systems (Table 1). Cardiac anomalies were the most common additional anomaly and affected 40% (*n* = 973) of infants with CDH, followed by nervous system (4.6%, *n* = 112) and chromosomal anomalies (4.5%, *n* = 110) (Table 2).

**TABLE 1:** Characteristics and birth prevalence of CDH cases per 10,000 livebirths

	No CDH	Any CDH		Isolated CDH		Complex CDH	
	No. infants (%)	No. infants (%)	Prevalence (95% CI)	No. infants (%)	Prevalence (95% CI)	No. infants (%)	Prevalence (95% CI)
Total	9,471,390	2435	2.6 (2.5, 2.7)	1285	1.4 (1.3, 1.4)	1150	1.2 (1.1, 1.3)
Infant birth period							
2002–2005	2,152,911 (22.7)	510 (20.9)	2.4 (2.2, 2.6)	332 (25.8)	1.5 (1.4, 1.7)	178 (15.5)	0.8 (0.7, 1.0)
2006–2009	2,417,348 (25.5)	598 (24.6)	2.5 (2.3, 2.7)	365 (28.4)	1.5 (1.4, 1.7)	233 (20.3)	1.0 (0.8, 1.1)
2010–2013	2,503,684 (26.4)	649 (26.7)	2.6 (2.4, 2.8)	320 (24.9)	1.3 (1.1, 1.4)	329 (28.6)	1.3 (1.2, 1.5)
2014–2017	2,397,447 (25.3)	678 (27.8)	2.8 (2.6, 3.0)	268 (20.9)	1.1 (1.0, 1.3)	410 (35.7)	1.7 (1.5, 1.9)
Infant sex							
Male	4,853,471 (51.2)	1465 (60.2)	3.0 (2.9, 3.2)	785 (61.1)	1.6 (1.5, 1.7)	680 (59.1)	1.4 (1.3, 1.5)
Female	4,609,449 (48.7)	968 (39.8)	2.1 (2.0, 2.2)	499 (38.8)	1.1 (1.0, 1.2)	469 (40.8)	1.0 (0.9, 1.1)
Unknown	8470 (0.1)	2 (0.1)	‐	1 (0.1)	‐	1 (0.1)	‐
Infant ethnicity							
White	6,083,478 (64.2)	1639 (67.3)	2.7 (2.6, 2.8)	884 (68.8)	1.5 (1.4, 1.6)	755 (65.7)	1.2 (1.2, 1.3)
South Asian	847,931 (9.0)	342 (14.0)	4.0 (3.6, 4.5)	149 (11.6)	1.8 (1.5, 2.1)	193 (16.8)	2.3 (2.0, 2.6)
Black	428,432 (4.5)	78 (3.2)	1.8 (1.4, 2.3)	39 (3.0)	0.9 (0.6, 1.2)	39 (3.4)	0.9 (0.6, 1.2)
Other	256,553 (2.7)	82 (3.4)	3.2 (2.5, 4.0)	42 (3.3)	1.6 (1.2, 2.2)	40 (3.5)	1.6 (1.1, 2.1)
Mixed	352,350 (3.7)	70 (2.9)	2.0 (1.5, 2.5)	45 (3.5)	1.3 (0.9, 1.7)	25 (2.2)	0.7 (0.5, 1.0)
Unknown	1,502,646 (15.9)	224 (9.2)	‐	126 (9.8)	‐	98 (8.5)	‐
Infant gestational age (weeks)							
Term (≥37)	7,207,994 (76.1)	1454 (59.7)	2.0 (1.9, 2.1)	786 (61.2)	1.1 (1.0, 1.2)	668 (58.1)	0.9 (0.9, 1.0)
Preterm (<37)	447,644 (4.7)	410 (16.8)	9.2 (8.3, 10.1)	187 (14.6)	4.2 (3.6, 4.8)	223 (19.4)	5.0 (4.3, 5.7)
Unknown	1,815,752 (19.2)	571 (23.4)	‐	312 (24.3)	‐	259 (22.5)	‐
Infant birthweight (g)							
Normal (≥2500)	7,639,978 (80.7)	1539 (63.2)	2.0 (1.9, 2.1)	871 (67.8)	1.1 (1.1, 1.2)	668 (58.1)	0.9 (0.8, 0.9)
Low (<2500)	452,585 (4.8)	463 (19.0)	10.2 (9.3, 11.2)	176 (13.7)	3.9 (3.3, 4.5)	287 (25.0)	6.3 (5.6, 7.1)
Unknown	1,378,827 (14.6)	433 (17.8)	‐	238 (18.5)	‐	195 (17.0)	‐
Maternal age (years)							
<20	502,198 (5.3)	133 (5.5)	2.6 (2.2, 3.1)	68 (5.3)	1.4 (1.1, 1.7)	65 (5.7)	1.3 (1.0, 1.6)
20–24	1,670,309 (17.6)	422 (17.3)	2.5 (2.3, 2.8)	223 (17.4)	1.3 (1.2, 1.5)	199 (17.3)	1.2 (1.0, 1.4)
25–34	5,255,260 (55.5)	1242 (51.0)	2.4 (2.2, 2.5)	661 (51.4)	1.3 (1.2, 1.4)	581 (50.5)	1.1 (1.0, 1.2)
35–39	1,501,096 (15.8)	400 (16.4)	2.7 (2.4, 2.9)	210 (16.3)	1.4 (1.2, 1.6)	190 (16.5)	1.3 (1.1, 1.5)
≥40	339,358 (3.6)	104 (4.3)	3.1 (2.5, 3.7)	50 (3.9)	1.5 (1.1, 1.9)	54 (4.7)	1.6 (1.2, 2.1)
Unknown	203,169 (2.1)	134 (5.5)	‐	73 (5.7)	‐	61 (5.3)	‐
Maternal deprivation							
Least deprived quintile	1,467,818 (15.5)	352 (14.5)	2.4 (2.2, 2.7)	198 (15.4)	1.3 (1.2, 1.6)	154 (13.4)	1.0 (0.9, 1.2)
4	1,531,746 (16.2)	328 (13.5)	2.1 (1.9, 2.4)	203 (15.8)	1.3 (1.1, 1.5)	125 (10.9)	0.8 (0.7, 1.0)
3	1,731,731 (18.3)	466 (19.1)	2.7 (2.5, 2.9)	257 (20.0)	1.5 (1.3, 1.7)	209 (18.2)	1.2 (1.0, 1.4)
2	2,084,053 (22.0)	535 (22.0)	2.6 (2.4, 2.8)	278 (21.6)	1.3 (1.2, 1.5)	257 (22.3)	1.2 (1.1, 1.4)
Most deprived quintile	2,625,322 (27.7)	743 (30.5)	2.8 (2.6, 3.0)	344 (26.8)	1.3 (1.2, 1.5)	399 (34.7)	1.5 (1.4, 1.7)
Unknown	30,720 (0.3)	11 (0.5)	‐	5 (0.4)	‐	6 (0.5)	‐
Maternal region							
London	1,787,986 (18.9)	450 (18.5)	2.5 (2.3, 2.8)	242 (18.8)	1.4 (1.2, 1.5)	208 (18.1)	1.2 (1.0, 1.3)
North East	436,869 (4.6)	145 (6.0)	3.3 (2.8, 3.9)	81 (6.3)	1.9 (1.5, 2.3)	64 (5.6)	1.5 (1.1, 1.9)
North West	1,251,348 (13.2)	331 (13.6)	2.6 (2.4, 2.9)	139 (10.8)	1.1 (0.9, 1.3)	192 (16.7)	1.5 (1.3, 1.8)
Yorkshire & Humber	939,193 (9.9)	245 (10.1)	2.6 (2.3, 3.0)	115 (8.9)	1.2 (1.0, 1.5)	130 (11.3)	1.4 (1.2, 1.6)
East Midlands	758,447 (8.0)	202 (8.3)	2.7 (2.3, 3.1)	117 (9.1)	1.5 (1.3, 1.8)	85 (7.4)	1.1 (0.9, 1.4)
West Midlands	1,019,576 (10.8)	300 (12.3)	2.9 (2.6, 3.3)	136 (10.6)	1.3 (1.1, 1.6)	164 (14.3)	1.6 (1.4, 1.9)
East of England	1,000,221 (10.6)	236 (9.7)	2.4 (2.1, 2.7)	142 (11.1)	1.4 (1.2, 1.7)	94 (8.2)	0.9 (0.8, 1.2)
South East	1,458,447 (15.4)	335 (13.8)	2.3 (2.1, 2.6)	197 (15.3)	1.4 (1.2, 1.6)	138 (12.0)	0.9 (0.8, 1.1)
South West	819,303 (8.7)	191 (7.8)	2.3 (2.0, 2.7)	116 (9.0)	1.4 (1.2, 1.7)	75 (6.5)	0.9 (0.7, 1.1)

Abbreviations: CDH, congenital diaphragmatic hernia; CI, confidence interval.

**TABLE 2:** Congenital anomalies outside of the respiratory and digestive systems among congenital diaphragmatic hernia cases

Congenital anomaly type	No. infants (%) (*N* = 2435)
Cardiac	973 (40.0)
Nervous system	112 (4.6)
Ear, face and neck	29 (1.2)
Orofacial	40 (1.6)
Genital	51 (2.1)
Urinary	102 (4.2)
Musculoskeletal	101 (4.2)
Chromosomal	110 (4.5)
Other	72 (3.0)

### 3.2 Birth prevalence of congenital diaphragmatic hernia subtypes

The overall birth prevalence of isolated and complex CDH were 1.4 (95% CI 1.3, 1.4) and 1.2 (95% CI 1.1, 1.3) per 10,000 livebirths, respectively (Table 1). Compared with infants born before 2010, those born later had a lower prevalence of isolated CDH but a higher prevalence of complex CDH. Both subtypes were more prevalent in males than females, with a male‐to‐female ratio of 3:2. Compared with white infants, both subtypes were less prevalent among Black infants, but only complex CDH was more prevalent among South Asian infants. Both subtypes were more prevalent among infants born preterm compared with infants born at or after term, with differences more pronounced for complex CDH.

Isolated CDH prevalence did not vary by maternal age, but complex CDH prevalence was greater for maternal age ≥ 40 years compared with 25–34 years (Table 1). Isolated CDH prevalence also did not vary by maternal deprivation whereas complex CDH was more prevalent among infants born to the most deprived mothers compared with the least deprived. Some regional variation in birth prevalence was observed for both subtypes. Compared with London, for example, isolated CDH birth prevalence was higher in the North East while complex CDH prevalence was higher in the North West and West Midlands.

### 3.3 Maternal age as a risk factor for occurrence of congenital diaphragmatic hernia subtypes

Neither univariable nor multivariable complete record analyses suggested an association between maternal age and isolated CDH (Table 3). Univariable analysis suggested an increased risk of complex CDH for maternal age ≥ 40 years compared with the reference group of 25–34 years. Maternal age < 20 years occurred more commonly in earlier study years when the birth prevalence of complex CDH was lower. Accounting for birth period increased the risk of complex CDH for maternal age < 20 years compared with the reference group. Compared with other ethnicities, South Asian infants were overrepresented in the reference age group but underrepresented among mothers aged <20 and ≥35 years. Accounting for ethnicity in addition to birth period increased the risk of complex CDH for maternal age < 20 years and 35–39 years.

**TABLE 3:** Risk of occurrence of CDH subtypes compared with no CDH, by maternal age

Maternal age (years)	Risk Ratio (95% confidence interval) for congenital diaphragmatic hernia subtypes by maternal age					
	Unadjusted	Accounting for birth period	Accounting for birth period and infant ethnicity	Accounting for birth period, infant ethnicity and deprivation	Accounting for birth period, infant ethnicity, deprivation and region	Excluding chromosomal anomalies and accounting for birth period, infant ethnicity, deprivation and region
Isolated CDH (*n* = 1093)						
<20	1.03 (0.79, 1.35)	0.98 (0.75, 1.28)	1.00 (0.76, 1.30)	1.00 (0.76, 1.31)	0.99 (0.76, 1.30)	‐
20–24	0.99 (0.85, 1.17)	0.97 (0.83, 1.15)	0.98 (0.83, 1.15)	0.98 (0.83, 1.15)	0.97 (0.83, 1.15)	‐
25–34	1.00 (Reference)	1.00 (Reference)	1.00 (Reference)	1.00 (Reference)	1.00 (Reference)	*‐*
35–39	1.09 (0.92, 1.28)	1.09 (0.92, 1.28)	1.10 (0.93, 1.30)	1.10 (0.94, 1.30)	1.11 (0.94, 1.30)	‐
≥40	1.11 (0.82, 1.51)	1.12 (0.82, 1.52)	1.15 (0.84, 1.56)	1.15 (0.84, 1.56)	1.15 (0.85, 1.56)	‐
Complex CDH (*n* = 993)^a^						
<20	1.23 (0.95, 1.61)	1.33 (1.02, 1.74)	1.44 (1.10, 1.88)	1.33 (1.01, 1.74)	1.31 (1.00, 1.72)	1.31 (0.99, 1.74)
20–24	1.05 (0.89, 1.25)	1.09 (0.91, 1.29)	1.11 (0.94, 1.32)	1.05 (0.88, 1.25)	1.04 (0.88, 1.24)	1.07 (0.89, 1.28)
25–34	1.00 (Reference)	1.00 (Reference)	1.00 (Reference)	1.00 (Reference)	1.00 (Reference)	1.00 (Reference)
35–39	1.18 (0.99, 1.39)	1.18 (0.99, 1.40)	1.21 (1.02, 1.44)	1.25 (1.05, 1.48)	1.26 (1.06, 1.49)	1.20 (1.00, 1.44)
≥40	1.48 (1.11, 1.98)	1.47 (1.11, 1.97)	1.54 (1.16, 2.06)	1.59 (1.19, 2.12)	1.61 (1.21, 2.15)	1.39 (1.00, 1.92)

*Note*: All analyses included infants with completely recorded infant and maternal characteristics.

Abbreviations: CDH, congenital diaphragmatic hernia; 95% CI, 95% confidence interval.

^a^ The analysis excluding chromosomal anomalies included 901 infants.

Accounting for deprivation and region attenuated the risk of complex CDH for maternal age < 20 years although an indication of increased risk remained. Conversely, after accounting for deprivation and region there was a slight increase in the risk of complex CDH among mothers aged 35–39 and ≥40 years. Results from multiple imputation analyses were in agreement although effect estimates were lower and evidence of an association between complex CDH and maternal age < 20 years was weaker (Table S5). Excluding infants with chromosomal anomalies in both complete record and multiple imputation analyses did not alter effect estimates for younger maternal age but reduced effect estimates for advanced maternal age, although there was still some weak evidence to suggest increased risk (Table 3 and Table S5).

### 3.4 Mortality of congenital diaphragmatic hernia subtypes in the first year of life

**FIGURE 2:**
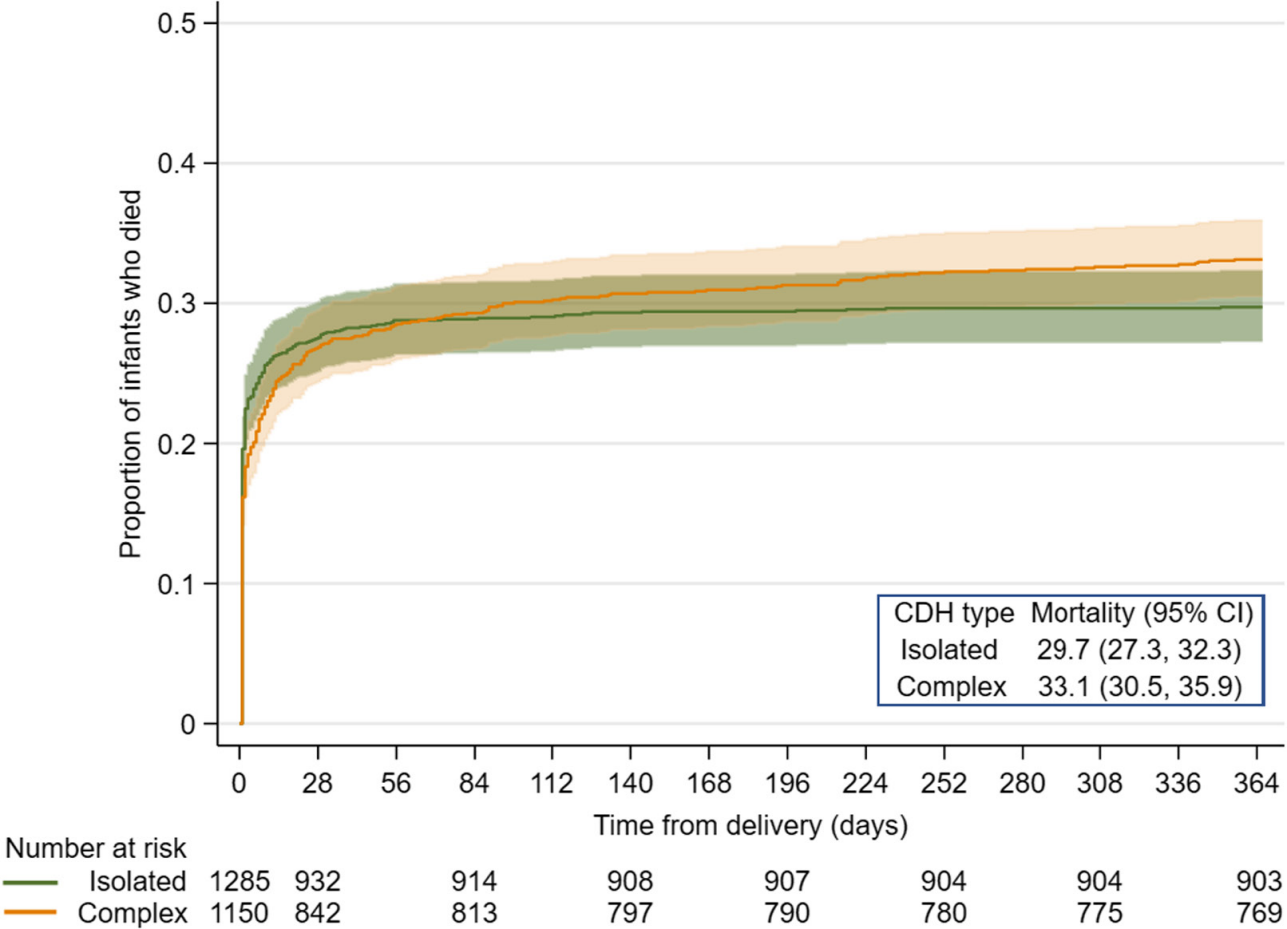
One‐year mortality by CDH subtype. CDH, congenital diaphragmatic hernia; CI, confidence interval

There were 34,235 deaths in the whole birth cohort during the first year of life; 1.1% (*n* = 382) occurred among infants with isolated CDH while a further 1.1% (*n* = 381) occurred among complex CDH cases. The overall 1‐year mortality rate for all livebirths with isolated CDH was 29.7% (95% CI 27.3, 32.3) and for complex CDH was 33.1% (95% CI 30.5, 35.9), with an indication of greater hazards in complex versus isolated CDH (HR 1.10, 95% CI 0.96, 1.27) (Figure 2). One‐year mortality estimates did not differ considerably by birth period or after including 26 additional deaths between the first and tenth birthday (Figures S1 and S2).

Mortality rates in the first year of life for CDH subtypes were highest around delivery (Figure 2). For both subtypes most deaths occurred among those without a surgical repair (Table 4). The crude proportion of infants with isolated CDH who died without repair was 27.3% (95% CI 24.9, 29.8) and was similar for complex CDH at 25.7% (95% CI 23.2, 28.3). Crude mortality after repair was markedly lower but differed between CDH subtypes. Just 2.4% of all infants with isolated CDH died following a repair (95% CI 1.6, 3.4) whereas this was three‐fold higher for complex CDH at 7.5% (95% CI 6.0, 9.2). The estimated HR for surgery during follow‐up for isolated CDH was 0.22 (95% CI 0.14, 0.34) and for complex CDH was 0.34 (95% CI 0.24, 0.47), indicating a strong protective effect of surgery in these children.

**TABLE 4:** Mortality and repairs of CDH subtypes in the first year of life

	Any CDH	Isolated CDH	Complex CDH
Total no. of infants	2435	1285	1150
Median no. days in hospital overall (IQR)	18 (6–38)	14 (4–28)	26 (9–53)
Median no. days in hospital during 1st continuous inpatient stay from birth (IQR)	17 (2–37)	13 (1–28)	23 (6–49)
No. of infants that died (%)	763 (31.3)	382 (29.7)	381 (33.1)
Median no. days from birth to death (IQR)	1 (0–8)	1 (0–2)	2 (0–16)
No. of infants that underwent surgical repair of CDH (%)	1676 (68.8)	868 (67.6)	808 (70.3)
Median no. days from birth to first repair (IQR)	4 (2–10)	4 (2–10)	5 (3–10)
No. infants without repair of CDH that died (%)	646 (26.5)	351 (27.3)	295 (25.7)
Median no. days from birth to death among those without repair (IQR)	1 (0–2)	1 (0–2)	1 (0–4)
No. infants without repair of CDH that did not die^a^ (%)	113 (4.6)	66 (5.1)	47 (4.1)
No. infants with repair of CDH that died (%)	117 (4.8)	31 (2.4)	86 (7.5)
Median no. days from birth to first repair among those who died (IQR)	6 (4–14)	5 (3–14)	6 (4–14)
Median no. days from first repair to death (IQR)	32 (9–78)	21 (7–52)	42 (10–87)
No. infants with repair of CDH that did not die (%)	1559 (64.0)	837 (65.1)	722 (62.8)
Median no. days from birth to initial repair among those who did not die (IQR)	4 (2–10)	4 (2–10)	5 (3–10)

*Note*: Proportions out of all infants in each column are shown and are crude estimates unadjusted for follow‐up time.

Abbreviations: CDH, congenital diaphragmatic hernia; IQR, interquartile range.

^a^ Of these 113 infants, 14 had repairs after the first year of life, ≤10 died after the first year of life, some may have died and the death certificates may have been delayed, and others may have been transferred to palliative care facilities not captured by Hospital Episode Statistics.

The proportion of infants that underwent repair was similar for both isolated and complex CDH (Table 4). For both subtypes, most initial repairs were carried out within 10 days of delivery and the proportions readmitted for further repair were also similar for isolated and for complex CDH. A small proportion of CDH cases had no repair and no death in the year after delivery for both isolated and for complex CDH.

### 3.5 Sensitivity analyses

Congenital diaphragmatic hernia cases with more complete records had broadly similar characteristics to the wider CDH population and a slight indication of lower mortality; however, similarities or differences in the mortality and repair of subtypes persisted (Tables S6–S9). Sensitivity analyses examining maternal age and CDH occurrence (restricted to infants with complete infant, maternal and birth characteristics) did not differ notably from the main analysis (restricted to infants with complete infant and maternal characteristics) except for increased risk of complex CDH among mothers aged <20 years compared with the reference group (Table S10). Effect estimates for this age group in the primary complete record analysis may have been biased upward compared with the wider CDH population, as partly indicated by the multiple imputation results (Table S5).

### 3.6 Comment

#### 3.6.1 Principal findings

Among a national cohort of 9.5 million livebirths, the prevalence of isolated CDH and complex CDH were 1.4 and 1.2 per 10,000 livebirths, respectively. The risk of complex but not isolated CDH was greater for maternal age ≥ 35 years (compared with 25–34 years). The association between advanced maternal age and complex CDH was largely explained by chromosomal anomalies. The 1‐year mortality rate was only slightly higher for complex compared with isolated CDH. Mortality for both subtypes was similar in the absence of surgery and in the neonatal period, when most deaths occurred, but was higher for complex than isolated CDH after surgery.

#### 3.6.2 Strengths of the study

A key strength of our study was the use of national, routinely‐collected administrative data. This enabled robust estimates of CDH prevalence and mortality among live‐born infants at a national scale. Our study avoided the bias associated with specialist registries that only include live‐born infants who attend tertiary centres and miss severely affected livebirths that die earlier. While population‐based regional registry studies can address this bias, these were only available for some areas in England until recently and studies were limited in size.^18^, ^19^, ^20^ Postnatal ascertainment of congenital anomalies by regional registries was variable when these studies occurred but has likely improved since 2015 when regional registries were incorporated into a national registry, which receives electronic notifications of cases from National Health Service Trusts.^28^, ^29^

#### 3.6.3 Limitations of the data

Severe CDH cases are more likely to result in fetal death or termination but could not be examined using HES as antenatal diagnoses are unreliably recorded and stillbirth certificates are not linked. Population‐based registry data on CDH cases in England during the study period suggested 3.9% were stillborn and 23.8% resulted in termination.^30^ Our birth prevalence and mortality estimates could be influenced by differential antenatal screening, antenatal diagnosis, termination and fetal death. We had no data on prenatal interventions such as fetoscopic endotracheal occlusion, size of defect or type of surgical approach and so could not assess how these might impact birth prevalence or mortality. While our estimates are useful for describing the burden of disease and overall prognosis of the livebirth population, reasons for any differences were not examined. For analyses examining maternal age and CDH occurrence, RRs could be biased downwards for older maternal ages which are associated with greater risk of fetal death and for younger maternal ages which are associated with higher rates of termination.^31^, ^32^

Incomplete data were a further limitation. Our findings from multiple imputation analyses and sensitivity analyses restricted to infants with completely recorded infant, maternal and birth characteristics suggest we cannot preclude the possibility that in the wider population of complex CDH (which includes infants with incomplete records), the risk of complex CDH is not increased for young maternal age. However, although infants with more complete records may represent a healthier subset of CDH infants, comparison of mortality or repairs between CDH subtypes produced similar results when restricted to infants with completely recorded characteristics.

#### 3.6.4 Interpretation

The prevalence estimate of any CDH was consistent with previous estimates, as were findings of a preponderance among males, preterm and low‐birthweight infants and findings that cardiac, nervous system and chromosomal anomalies were among the most frequent additional anomalies.^2^, ^7^, ^33^, ^34^ The proportion of CDH cases identified as complex in our study was higher than in registry data which may not capture additional anomalies among early deaths or diagnosed after the neonatal period.^17^, ^18^, ^19^, ^20^, ^35^ In line with the known prognosis, approximately one‐third of infants with CDH died in the first year of life and few deaths occurred beyond this.^17^, ^20^, ^36^, ^37^, ^38^, ^39^

Birth prevalence of complex CDH increased over time, consistent with a European registry study.^34^ While this could represent a true increase in occurrence, it could also be driven by improved diagnosis or recording of additional anomalies. Compared with white infants in our study, complex but not isolated CDH was more prevalent among South Asian infants while both subtypes were less prevalent among Black infants which is consistent with previous studies.^1^, ^2^ Ethnic differences in the birth prevalence of congenital anomalies may be underpinned by deprivation, health inequities including barriers to reproductive care or missed diagnoses and cultural or community influences. Individual‐level deprivation data were not available but both subtypes were more prevalent in regions associated with greater levels of deprivation,^40^ and complex CDH was also more prevalent among mothers in the most deprived small‐area‐level quintile.

Advanced maternal age was not associated with isolated CDH but was associated with complex CDH, which suggested different mechanisms of occurrence. The genetics of isolated CDH remain elusive but studies report it is more likely than complex CDH to be associated with inherited, non‐age‐dependent genetic variants.^41^ The increased risk of complex CDH for advanced maternal age was reduced after excluding age‐dependent chromosomal anomalies, consistent with their known role in CDH aetiology.^1^ The remaining association between advanced maternal age and complex CDH may be explained by unexamined factors including de novo age‐dependent genetic abnormalities and other age‐dependent factors thought to be associated with congenital anomaly occurrence such as parity, maternal chronic conditions and obesity.^42^, ^43^, ^44^ There was some weak evidence of an elevated risk of complex CDH for young maternal age. While we accounted for area‐level deprivation and region these indicators do not capture all dimensions of deprivation and so residual social disadvantage could allow risk to remain elevated. Our results are consistent with previous suggestions of multiple causal mechanisms for CDH.^1^, ^2^

UK studies that compared 1‐year mortality for isolated and complex CDH reported conflicting results.^17^, ^18^, ^19^, ^20^ We found 1‐year mortality rates for complex CDH appeared only slightly higher than isolated CDH and remained stable during the study period. One‐year mortality rates concealed that mortality was greater among complex cases after surgical repair or after the neonatal period (when most repairs had taken place). Most deaths occurred among infants without repair and affected similar proportions of each subtype. We found a higher proportion of deaths before surgical repair than previously estimated using data from surgical centres which miss deaths before transfer for surgery.^17^

## 4 CONCLUSIONS

We quantified the national prevalence of isolated and complex CDH among livebirths and described differences in their occurrence and prognosis. Young and advanced maternal age were associated with an increased risk of complex but not isolated CDH, with chromosomal anomalies accounting for some of the increased risk associated with advanced maternal age. The prognosis of subtypes differed after surgical repair, with complex cases experiencing higher mortality rates. Administrative data could be usefully enhanced through linkage to specialist registry data (containing information on genetic abnormalities, antenatal diagnoses, disease severity, prenatal interventions and postnatal management) in order to help further our understanding of the prevalence, aetiology and prognosis of subtypes.

## AUTHOR CONTRIBUTIONS

MP, BLD, SL, RG and PDC conceptualised and designed the study. MP and SL carried out initial analyses. MP, BLD, SL, AZ, RG and PDC contributed to the interpretation of the data. MP drafted the manuscript. All authors critically reviewed the manuscript for important intellectual content. All authors approved the final manuscript as submitted and agree to be accountable for all aspects of the work.

## FUNDING INFORMATION

This project is funded by the National Institute for Health Research Great Ormond Street Hospital Biomedical Research Centre (NIHR GOSH‐BRC). NIHR GOSH‐BRC had no role in the design, conduct, analysis or interpretation of the study and no role in drafting the manuscript.

## CONFLICT OF INTEREST

The authors have no conflicts of interest to disclose.

## Supporting information


Appendix S1.

## Data Availability

Source data can be accessed by application to NHS Digital (https://digital.nhs.uk/services/data‐access‐request‐service‐dars).
