# Supplementary material for: Congenital diaphragmatic hernia subtypes: Comparing birth prevalence, occurrence by maternal age, and mortality in a national birth cohort
Source: Paediatr Perinat Epidemiol. 2022 Nov 28;37(2):143–53. doi: 10.1111/ppe.12939 (PMC10099870; doi:10.1111/ppe.12939)
Supplement: Supplementary file 1 — Appendix S1. [file PPE-37-143-s001.docx]

**Supplementary Material**

**Supplementary Box 1 - Further details on methods**

*Model specification for the analyses of maternal age and occurrence of CDH subtypes*

The main exposure variable, maternal age, was treated in the main analyses as a categorical variable (with 5 levels: <20, 20-24, 25-34, 35-39, and ≥40 years) for ease of interpretation. However, we also fitted various models using the original variable on a continuous scale, including it as a polynomial function (with age, age squared and age cubic) or using fractional polynomials. In the first case, a linear relationship with maternal age fitted the data best according to the likelihood ratio test, with an estimated RR per 1 year increase in maternal age equal to 1.02 (95% CI: 0.99, 1.05), while the fractional polynomial specification indicated a fairly linear relationship but with an increasing slope beyond age 45:


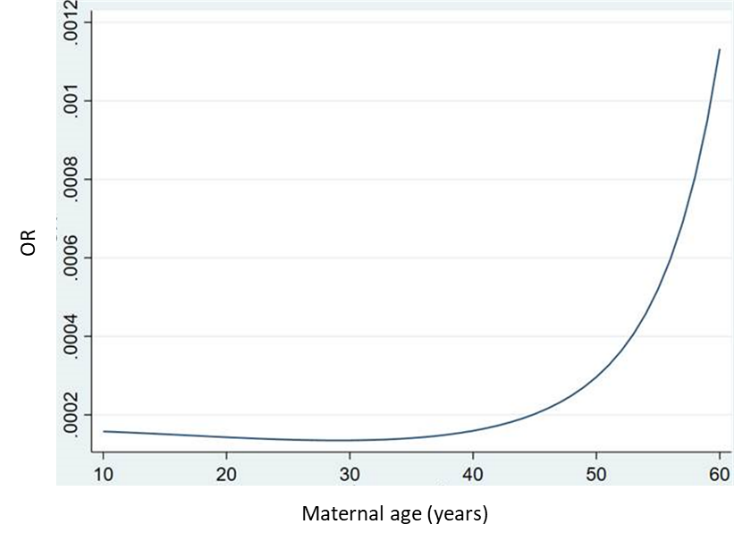


In addition to maternal age the model also included birth period, infant ethnicity, maternal deprivation, and region. Region was included as a covariate and not treated as a clustering variable in a multilevel model because the total number of regions in England (nine) is too small to estimate the between region-variance with sufficient precision. Allowing for fixed effects, that is constant differences across regions estimated by dummy indicators, is generally preferable (Rabe-Hesketh and Skrondal, Section 2.8 “Multilevel and Longitudinal Modelling using Stata” 2012, Stata Press).

To deal with the loss of records due to missing information on maternal age and/or infant ethnicity and maternal deprivation we used multiple imputation, with 10 imputed sets generated using chained equations. The imputation model included all variables involved in the model examining maternal age and CDH subtypes (maternal age, birth period, infant ethnicity, maternal deprivation, region, CDH and any other congenital anomaly). The model also included gestational age and birth weight as missingness in these variables was correlated with missingness of maternal age.

**Supplementary Table 1 – Diagnosis and procedure codes used to identify evidence of congenital diaphragmatic hernia.**

| **ICD-10 diagnosis codes Fields searched** | | |
| --- | --- | --- |
| Q790 | Congenital diaphragmatic hernia | All diagnosis fields in hospital admission records during the first year of life.  All cause of death fields in Office for National Statistics death registrations for those aged ≤1 years at the time of death. |
| **OPCS-4 procedure codes Fields searched** | | |
| G232 | Repair of diaphragmatic hernia using thoracic approach NEC | All procedure fields in hospital admission records during the first year of life. |
| G234 | Repair of diaphragmatic hernia using abdominal approach NEC |  |
| G238 | Other specified repair of diaphragmatic hernia |  |
| G239 | Unspecified repair of diaphragmatic hernia |  |
| T161 | Insertion of prosthesis for repair of diaphragm |  |
| T164 | Repair of congenital diaphragmatic hernia |  |
| T165 | Suture of diaphragm NEC |  |

**Supplementary Table 2 – Diagnosis and procedure codes searched for to provide supportive evidence of congenital diaphragmatic hernia.**

| **Codes** | | **Fields searched** |
| --- | --- | --- |
| **ICD-10 diagnosis codes for lung hypoplasia** | | |
| Q336 | Hypoplasia and dysplasia of lung | All diagnoses in hospital admission records; All causes of death in Office for National Statistics death registrations |
| **ICD-10 diagnosis codes for respiratory distress** | | |
| J96 | Respiratory failure, not elsewhere classified | All diagnoses in hospital admission records that occurred in the same continuous inpatient stay as the CDH repair in the first year of life^a^ |
| P22 | Respiratory distress of new-born |  |
| P282 | Cyanotic attacks of new-born |  |
| P284 | Other apnoea of new-born |  |
| P285 | Respiratory failure of new-born |  |
| Z991 | Dependence on respirator |  |
| R092 | Respiratory arrest |  |
| R068 | Other and unspecified abnormalities of breathing |  |
| **ICD-10 diagnosis codes indicating pulmonary hypertension** | | |
| I270 | Primary pulmonary hypertension | All diagnoses in hospital admission records that occurred in the same continuous inpatient stay as the CDH repair in the first year of life^a^ |
| I272 | Other secondary pulmonary hypertension |  |
| P293 | Persistent fetal circulation |  |
| P292 | Neonatal hypertension |  |
| **ICD-10 diagnosis codes for hypoxia/asphyxia** | | |
| P201 | Intrauterine hypoxia first noted during labour and delivery | All diagnoses in hospital admission records that occurred from delivery to the end of the first inpatient stay and which co-occurred with the CDH repair^b^ |
| P209 | Intrauterine hypoxia, unspecified |  |
| P21 | Birth asphyxia |  |
| **ICD-10 diagnosis codes indicating tracheostomy** | | |
| Z430 | Attention to tracheostomy | All diagnoses in hospital admission records that occurred from delivery to the end of the first inpatient stay and which co-occurred with the CDH repair^b^ |
| J950 | Tracheostomy malfunction |  |
| Z930 | Tracheostomy status |  |
| **OPCS-4 procedure codes indicating invasive ventilation** | | |
| E423 | Temporary tracheostomy | All procedures in hospital admission records that occurred from delivery to the end of the first inpatient stay and which co-occurred with the CDH repair^b^ |
| E424 | Revision of tracheostomy |  |
| E425 | Closure of tracheostomy |  |
| E426 | Replacement of tracheostomy tube |  |
| E427 | Removal of tracheostomy tube |  |
| E851 | Invasive ventilation |  |
| X561 | Nasotracheal intubation |  |
| X562 | Endotracheal intubation |  |
| X569 | Unspecified intubation of trachea |  |
| X581 | Extracorporeal membrane oxygenation |  |

**^a^**All continuous inpatient stays in the first year of life which involved a CDH repair were searched to account for late-presenting infants with symptoms of respiratory distress and pulmonary hypertension. ^b^The first continuous inpatient stay (from delivery to final discharge) which involved a CDH repair was searched to identify infants presenting with conditions typically associated with a diagnosis of CDH at birth (respiratory distress, hypoxia/asphyxia, hypertension) or those with clinical management indicative of CDH identified at birth (invasive ventilation).

**Supplementary Table 3 – Diagnosis codes used to exclude infants.**

| **ICD-10 diagnosis codes** | **Fields searched** |
| --- | --- |
| Q401 Hiatus hernia^a^  Q792 Exomphalos  Q793 Gastroschisis  Q39 Oesophageal malformations | All diagnosis fields in hospital admission records.  All cause of death fields in Office for National Statistics death registrations. |

**^a^**Infants were excluded if they had evidence of a hiatal hernia and none of the following: CDH recorded on the death certificate, diagnosis of lung hypoplasia at any time, an indication in the delivery record or in any record containing a CDH diagnosis or repair of respiratory distress, hypoxia/asphyxia or invasive ventilation. Respiratory distress, hypoxia/asphyxia and invasive ventilation (including tracheostomy) were defined using the codes in Supplementary Table 2.

**Supplementary Table 4 – Diagnosis codes used to identify evidence of additional malformations**^a^

| **ICD-10 diagnosis codes** | |
| --- | --- |
| **Nervous system malformations** | |
| Q00 | Anencephaly and similar malformations |
| Q01 | Encephalocele |
| Q02 | Microcephaly |
| Q03 | Congenital hydrocephalus |
| Q04 | Other congenital malformations of brain |
| Q05 | Spina bifida |
| Q06 | Other congenital malformations of spinal cord |
| Q07 | Other congenital malformations of nervous system |
| **Ear, face and neck malformations** | |
| Q104 | Absence and agenesis of lacrimal apparatus |
| Q107 | Congenital malformations of orbit |
| Q11 | Anophthalmos, microphthalmos and macrophthalmos |
| Q12 | Congenital lens malformations |
| Q130 | Coloboma of iris |
| Q131 | Absence of iris |
| Q132 | Other congenital malformations of iris |
| Q133 | Congenital corneal opacity |
| Q134 | Other congenital corneal malformations |
| Q138 | Other congenital malformations of anterior segment of eye |
| Q139 | Congenital malformations of anterior segment of eye, unspecified |
| Q14 | Congenital malformations of posterior segment of eye |
| Q15 | Other congenital malformations of eye |
| Q16 | Congenital malformations of ear causing hearing impairment |
| Q188 | Other specified congenital malformations of face and neck |
| **Cardiac malformations** | |
| Q20 | Congenital malformations of cardiac chambers and connections |
| Q21 | Congenital malformations of cardiac septa |
| Q22 | Congenital malformations of pulmonary and tricuspid valves |
| Q23 | Congenital malformations of aortic and mitral valves |
| Q24 | Other congenital malformations of heart |
| Q25 | Congenital malformations of great arteries |
| Q26 | Congenital malformations of great veins |
| **Orofacial malformations** | |
| Q35 | Cleft palate |
| Q36 | Cleft lip |
| Q37 | Cleft palate with cleft lip |
| **Genital malformations** | |
| Q500 | Congenital absence of ovary |
| Q51 | Congenital malformations of uterus and cervix |
| Q520 | Congenital absence of vagina |
| Q521 | Doubling of vagina |
| Q522 | Congenital rectovaginal fistula |
| Q524 | Other congenital malformations of vagina |
| Q540 | Hypospadias, balanic |
| Q541 | Hypospadias, penile |
| Q542 | Hypospadias, penoscrotal |
| Q543 | Hypospadias, perineal |
| Q548 | Other hypospadias |
| Q549 | Hypospadias, unspecified |
| Q550 | Absence and aplasia of testis |
| Q555 | Congenital absence and aplasia of penis |
| Q56 | Indeterminate sex and pseudohermaphroditism |
| **Urinary malformations** | |
| Q601 | Renal agenesis, bilateral |
| Q602 | Renal agenesis, unspecified |
| Q604 | Renal hypoplasia, bilateral |
| Q605 | Renal hypoplasia, unspecified |
| Q606 | Potter syndrome |
| Q61 | Cystic kidney disease |
| Q620 | Congenital hydronephrosis |
| Q621 | Atresia and stenosis of ureter |
| Q622 | Congenital megaloureter |
| Q623 | Other obstructive defects of renal pelvis and ureter |
| Q624 | Agenesis of ureter |
| Q625 | Duplication of ureter |
| Q626 | Malposition of ureter |
| Q628 | Other congenital malformations of ureter |
| Q630 | Accessory kidney |
| Q631 | Lobulated, fused and horseshoe kidney |
| Q632 | Ectopic kidney |
| Q638 | Other specified congenital malformations of kidney |
| Q639 | Congenital malformation of kidney, unspecified |
| Q64 | Other congenital malformations of urinary system |
| **Musculoskeletal malformations** | |
| Q650 | Congenital dislocation of hip, unilateral |
| Q651 | Congenital dislocation of hip, bilateral |
| Q652 | Congenital dislocation of hip, unspecified |
| Q658 | Other congenital deformities of hip |
| Q659 | Congenital deformity of hip, unspecified |
| Q675 | Congenital deformity of spine |
| Q682 | Congenital deformity of knee |
| Q683 | Congenital bowing of femur |
| Q684 | Congenital bowing of tibia and fibula |
| Q685 | Congenital bowing of long bones of leg, unspecified |
| Q71 | Reduction defects of upper limb |
| Q72 | Reduction defects of lower limb |
| Q73 | Reduction defects of unspecified limb |
| Q74 | Other congenital malformations of limb(s) |
| Q750 | Craniosynostosis |
| Q751 | Craniofacial dysostosis |
| Q753 | Macrocephaly |
| Q754 | Mandibulofacial dysostosis |
| Q755 | Oculomandibular dysostosis |
| Q758 | Other specified congenital malformations of skull and face bones |
| Q759 | Congenital malformation of skull and face bones, unspecified |
| Q761 | Klippel-Feil syndrome |
| Q762 | Congenital spondylolisthesis |
| Q763 | Congenital scoliosis due to congenital bony malformation |
| Q764 | Other congenital malformations of spine, not associated with scoliosis |
| Q77 | Osteochondrodysplasia with defects of growth of tubular bones and spine |
| Q78 | Other osteochondrodysplasias |
| Q794 | Prune belly syndrome |
| Q795 | Other congenital malformations of abdominal wall |
| Q796 | Ehlers-Danlos syndrome |
| Q798 | Other congenital malformations of the musculoskeletal system |
| **Other malformations** | |
| Q820 | Hereditary lymphoedema |
| Q821 | Xeroderma pigmentosum |
| Q822 | Mastocytosis |
| Q823 | Incontinentia pigmenti |
| Q824 | Ectodermal dysplasia (anhidrotic) |
| Q829 | Congenital malformation of skin, unspecified |
| Q85 | Phakomatoses, not elsewhere classified |
| Q860 | Fetal alcohol syndrome (dysmorphic) |
| Q861 | Fetal hydantoin syndrome |
| Q862 | Dysmorphism due to warfarin |
| Q868 | Other congenital malformation syndromes due to known exogenous causes |
| Q878 | Other specified congenital malformation syndromes, not elsewhere classified |
| Q891 | Congenital malformations of adrenal gland |
| Q892 | Congenital malformations of other endocrine glands |
| Q893 | Situs inversus |
| Q894 | Conjoined twins |
| Q897 | Multiple congenital malformations, not elsewhere classified |
| Q898 | Other specified congenital malformations |
| Q899 | Congenital malformation, unspecified |
| **Chromosomal** | |
| Q90 | Down syndrome |
| Q91 | Edward syndrome and Patau syndrome |
| Q92 | Other trisomies and partial trisomies of the autosomes, not elsewhere classified |
| Q93 | Monosomies and deletions from the autosomes, not elsewhere classified |
| Q952 | Balanced autosomal rearrangement in abnormal individual |
| Q953 | Balanced sex/autosomal rearrangement in abnormal individual |
| Q97 | Other sex chromosome abnormalities, female phenotype, not elsewhere classified |
| Q99 | Other chromosome abnormalities, not elsewhere classified |

^a^All diagnosis fields in hospital admission records during the first year of life and all cause of death fields in Office for National Statistics death registrations for those aged ≤1 years at the time of death were searched for the codes above.

|  | **Risk Ratios for CDH subtypes by maternal age (95% CI)** | | | | | |
| --- | --- | --- | --- | --- | --- | --- |
| **Maternal age (years)** | Univariable | Accounting for  birth period | Accounting for  birth period & ethnicity | Accounting for  birth period, ethnicity & deprivation | Accounting for  birth period, ethnicity, deprivation & region | Excluding chromosomal anomalies & accounting for  birth period, ethnicity, deprivation & region |
| Isolated CDH | N=1,285 | N=1,285 | N=1,285 | N=1,285 | N=1,285 | - |
| <20 | 1.06 (0.82,1.36) | 1.02 (0.79,1.31) | 1.03 (0.80,1.33) | 1.04 (0.81,1.34) | 1.04 (0.80,1.34) | - |
| 20-24 | 1.06 (0.91,1.24) | 1.05 (0.90,1.22) | 1.05 (0.90,1.23) | 1.06 (0.90,1.24) | 1.05 (0.90,1.23) | - |
| 25-34 | *1.00 (Reference)* | *1.00 (Reference)* | *1.00 (Reference)* | *1.00 (Reference)* | *1.00 (Reference)* | ***-*** |
| 35-39 | 1.11 (0.95,1.30) | 1.11 (0.95,1.30) | 1.12 (0.95,1.31) | 1.12 (0.95,1.31) | 1.12 (0.95,1.31) | - |
| ≥40 | 1.16 (0.86-1.54) | 1.16 (0.87,1.55) | 1.18 (0.88,1.58) | 1.18 (0.88,1.58) | 1.18 (0.88,1.58) | - |
| Complex CDH | N=1,150 | N=1,150 | N=1,150 | N=1,150 | N=1,150 | N=1,040 |
| <20 | 1.16 (0.90,1.50) | 1.27 (0.98,1.64) | 1.36 (1.05,1.76) | 1.24 (0.96,1.61) | 1.23 (0.95,1.60) | 1.24 (0.94,1.62) |
| 20-24 | 1.07 (0.91,1.26) | 1.11 (0.94,1.31) | 1.13 (0.96,1.34) | 1.07 (0.90,1.26) | 1.06 (0.90,1.25) | 1.09 (0.92,1.29) |
| 25-34 | *1.00 (Reference)* | *1.00 (Reference)* | *1.00 (Reference)* | *1.00 (Reference)* | *1.00 (Reference)* | *1.00 (Reference)* |
| 35-39 | 1.14 (0.97,1.33) | 1.14 (0.97,1.33) | 1.16 (0.99,1.37) | 1.20 (1.02,1.41) | 1.21 (1.03,1.42) | 1.16 (0.98,1.38) |
| ≥40 | 1.43 (1.09,1.89) | 1.42 (1.07,1.87) | 1.47 (1.12,1.94) | 1.52 (1.15,2.00) | 1.54 (1.16,2.03) | 1.34 (0.98-1.84) |

**Supplementary Table 5 – Risk of occurrence of CDH subtypes compared with no CDH, by maternal age**

Analyses included all infants after using multiple imputation to estimate values for missing infant and maternal characteristics. Abbreviations: CDH, congenital diaphragmatic hernia; 95% CI, 95% confidence interval

**Supplementary Table 6 – Characteristics of infants with varying completeness of records^a^.**

|  | **No CDH** | | **Isolated CDH** | | **Complex CDH** | |
| --- | --- | --- | --- | --- | --- | --- |
|  | % of infants with complete recording of infant & maternal characteristics  (N=7,804,224) | % of infants with complete recording of infant, maternal & birth characteristics  (N=6,448,975) | % of infants with complete recording of infant & maternal characteristics  (N=1,093) | % of infants with complete recording of infant, maternal & birth characteristics (N=869) | % of infants with complete recording of infant & maternal characteristics  (N=993) | % of infants with complete recording of infant, maternal & birth characteristics (N=791) |
| **Infant birth period** | |  |  |  |  |  |
| 2002-2005 | 17.6 | 14.9 | 22.9 | 20.7 | 13.4 | 11.1 |
| 2006-2009 | 25.2 | 22.9 | 28.6 | 27.4 | 18.9 | 17.8 |
| 2010-2013 | 29.3 | 32.3 | 26.2 | 28.3 | 30.4 | 31.0 |
| 2014-2017 | 28.0 | 30.0 | 22.4 | 23.6 | 37.3 | 40.1 |
| **Infant sex** |  |  |  |  |  |  |
| Male | 51.4 | 51.4 | 61.2 | 61.8 | 60.1 | 60.6 |
| Female | 48.5 | 48.6 | 38.8 | 38.2 | 39.9 | 39.4 |
| Unknown | 0.1 | 0.0 | 0.0 | 0.0 | 0.0 | 0.0 |
| **Infant ethnicity** |  |  |  |  |  |  |
| White | 76.3 | 76.4 | 76.0 | 77.6 | 71.9 | 71.2 |
| South Asian | 10.7 | 10.7 | 13.1 | 12.2 | 17.9 | 18.8 |
| Black | 5.4 | 5.2 | 3.4 | 3.1 | 3.8 | 4.2 |
| Other | 3.2 | 3.2 | 3.7 | 3.6 | 3.8 | 3.4 |
| Mixed | 4.4 | 4.4 | 3.8 | 3.6 | 2.5 | 2.4 |
| Unknown | 0.0 | 0.0 | 0.0 | 0.0 | 0.0 | 0.0 |
| **Infant gestational age (weeks)** | |  |  |  |  |  |
| Term (≥37) | 78.9 | 94.1 | 67.1 | 81.1 | 62.4 | 75.9 |
| Preterm (<37) | 5.0 | 5.9 | 15.7 | 18.9 | 20.3 | 24.1 |
| Unknown | 16.1 | 0.0 | 17.2 | 0.0 | 17.2 | 0.0 |
| **Infant birthweight (g)** | |  |  |  |  |  |
| Normal (≥2500) | 83.1 | 94.4 | 72.6 | 84.5 | 62.0 | 70.8 |
| Low (<2500) | 5.0 | 5.6 | 14.2 | 15.5 | 25.7 | 29.2 |
| Unknown | 12.0 | 0.0 | 13.2 | 0.0 | 12.3 | 0.0 |
| **Maternal age (years)** | |  |  |  |  |  |
| <20 | 5.3 | 5.2 | 5.4 | 5.4 | 6.1 | 6.7 |
| 20-24 | 18.0 | 18.0 | 17.6 | 18.0 | 17.7 | 16.7 |
| 25-34 | 56.8 | 57.0 | 55.7 | 56.0 | 53.2 | 54.0 |
| 35-39 | 16.2 | 16.1 | 17.3 | 16.7 | 17.8 | 17.4 |
| ≥40 | 3.7 | 3.7 | 4.0 | 3.9 | 5.1 | 5.2 |
| Unknown | 0.0 | 0.0 | 0.0 | 0.0 | 0.0 | 0.0 |
| **Maternal deprivation** | |  |  |  |  |  |
| Q5: Least deprived | 15.2 | 15.1 | 15.2 | 15.2 | 13.6 | 13.5 |
| Q4 | 16.0 | 15.9 | 15.5 | 15.9 | 11.3 | 10.5 |
| Q3 | 18.3 | 18.3 | 19.5 | 18.9 | 18.1 | 17.6 |
| Q2 | 22.3 | 22.1 | 22.2 | 22.7 | 22.1 | 22.1 |
| Q1: Most deprived | 28.2 | 28.7 | 27.6 | 27.4 | 34.9 | 36.3 |
| Unknown | 0.0 | 0.0 | 0.0 | 0.0 | 0.0 | 0.0 |
| **Maternal region** |  |  |  |  |  |  |
| London | 19.0 | 18.6 | 18.6 | 18.6 | 17.3 | 15.9 |
| North East | 5.1 | 5.3 | 7.0 | 7.6 | 5.9 | 6.2 |
| North West | 12.9 | 13.6 | 11.3 | 11.9 | 16.8 | 16.8 |
| Yorkshire & Humber | 10.5 | 10.2 | 9.1 | 7.9 | 11.1 | 12.1 |
| East Midlands | 7.5 | 7.8 | 8.6 | 8.9 | 7.0 | 6.8 |
| West Midlands | 11.0 | 11.2 | 10.7 | 10.8 | 15.0 | 16.4 |
| East of England | 10.5 | 10.1 | 10.7 | 10.5 | 8.1 | 6.6 |
| South East | 14.8 | 14.4 | 14.7 | 13.5 | 11.9 | 11.8 |
| South West | 8.7 | 8.9 | 9.2 | 10.4 | 7.0 | 7.3 |

^a^We examined infants with complete recording of infant and maternal characteristics as well as infants with complete recording of infant, maternal, and birth characteristics. Abbreviations: CDH, congenital diaphragmatic hernia

**Supplementary Table 7 – Comparison of congenital anomalies outside of the respiratory and digestive systems among CDH cases with varying completeness of records.**

| **Congenital anomaly type** | **All infants**  **(N=2,435)** | **Infants with completely recorded infant and maternal characteristics**  **(N=2086)** | **Infants with completely recorded infant, maternal and birth characteristics**  **(N=1,660)** |
| --- | --- | --- | --- |
| Cardiac | 973 (40.0) | 852 (40.8) | 685 (41.3) |
| Nervous system | 112 (4.6) | 95 (4.6) | 78 (4.7) |
| Ear, face & neck | 29 (1.2) | 24 (1.2) | 15 (0.9) |
| Orofacial | 40 (1.6) | 34 (1.6) | 26 (1.6) |
| Genital | 51 (2.1) | 41 (2.0) | 28 (1.7) |
| Urinary | 102 (4.2) | 83 (4.0) | 66 (4.0) |
| Musculoskeletal | 101 (4.2) | 90 (4.3) | 69 (4.2) |
| Chromosomal | 110 (4.5) | 92 (4.4) | 69 (4.2) |
| Other | 72 (3.0) | 59 (2.8) | 49 (3.0) |

Abbreviations: CDH, congenital diaphragmatic hernia

**Supplementary Table 8 – Comparison of mortality and repairs of CDH subtypes in the first year of life among CDH cases with varying completeness of records.**

|  |  | **All infants** | |  | **Infant with complete recording of infant & maternal characteristics** | |  | **Infants with complete recording of infant, maternal & birth characteristics** | |
| --- | --- | --- | --- | --- | --- | --- | --- | --- | --- |
|  |  | Isolated  CDH | Complex CDH |  | Isolated  CDH | Complex  CDH |  | Isolated  CDH | Complex  CDH |
| Total no. of infants |  | 1,285 | 1,150 |  | 1,093 | 993 |  | 869 | 791 |
| Median no. days in hospital overall (IQR) |  | 14 (4-28) | 26 (9-53) |  | 15 (6-28) | 28 (12-56) |  | 15 (7-29) | 28 (12-57) |
| Median no. days in hospital during 1^st^ continuous inpatient stay from birth (IQR) |  | 13 (1-28) | 23 (6-49) |  | 13 (2-28) | 25 (9-51) |  | 14 (2-28) | 25 (9-53) |
| No. of infants that died (%) |  | 382 (29.7) | 381 (33.1) |  | 290 (26.5) | 291 (29.3) |  | 223 (25.7) | 231 (29.2) |
| Median no. days from birth to death (IQR) |  | 1 (0-2) | 2 (0-16) |  | 1 (0-3) | 2 (0-22) |  | 1 (1-4) | 2 (0-18) |
|  |  |  |  |  |  |  |  |  |  |
| No. of infants that underwent surgical repair of CDH (%) |  | 868 (67.6) | 808 (70.3) |  | 775 (70.9) | 733 (73.8) |  | 628 (72.3) | 580 (73.3) |
| Median no. days from birth to first repair (IQR) |  | 4 (2-10) | 5 (3-10) |  | 4 (2-11) | 5 (3-10) |  | 4 (2-14) | 5 (3-10) |
|  |  |  |  |  |  |  |  |  |  |
| No. infants without repair of CDH that died (%) |  | 351 (27.3) | 295 (25.7) |  | 264 (24.2) | 218 (22.0) |  | 202 (23.2) | 178 (22.5) |
| No. infants with repair of CDH that died (%) |  | 31 (2.4) | 86 (7.5) |  | 26 (2.4) | 73 (7.4) |  | 21 (3.3) | 53 (9.1) |

Proportions out of all infants in each column are shown and are crude estimates unadjusted for follow-up time. Abbreviations: CDH, congenital diaphragmatic hernia; IQR, interquartile range.

**Supplementary Table 9 – Comparison of one-year mortality estimates by CDH subtypes among cases with varying completeness of records.**

|  | **All infants** | | **Infants with complete recording of infant and maternal characteristics** | | **Infants with complete recording of infant, maternal and birth characteristics** | |
| --- | --- | --- | --- | --- | --- | --- |
|  | **n** | **% died (95% CI)** | **n** | **% died (95% CI)** | **n** | **% died (95% CI)** |
| Isolated | 1,285 | 29.7 (27.3,32.3) | 1093 | 26.5 (24.0,29.3) | 869 | 25.7 (22.9,28.7) |
| Complex | 1,150 | 33.1 (30.5,35.9) | 993 | 29.3 (26.6,32.2) | 791 | 29.2 (26.2,32.5) |

Abbreviations: CDH, congenital diaphragmatic hernia

**Supplementary Table 10 – Comparison of main and sensitivity analyses examining the risk of CDH subtypes compared with no CDH, by maternal age.**

|  | **Risk Ratios for CDH subtypes by maternal age (95% CI)** | |
| --- | --- | --- |
| **Maternal age (years)** | Main analysis including all infants after accounting for birth period, ethnicity, maternal deprivation & region^a^ | Sensitivity analysis including only infants with complete recording of all covariates after accounting for birth period, ethnicity, maternal deprivation & region^b^ |
| Isolated CDH | N=1093 | N=869 |
| <20 | 0.99 (0.76,1.30) | 1.02 (0.75,1.38) |
| 20-24 | 0.97 (0.83,1.15) | 1.00 (0.83,1.20) |
| 25-34 | *1.00 (Reference)* | *1.00 (Reference)* |
| 35-39^a^ | 1.11 (0.94,1.30) | 1.07 (0.90,1.27) |
| ≥40^a^ | 1.15 (0.85,1.56) |  |
| Complex CDH | N=993 | N=791 |
| <20 | 1.31 (1.00,1.72) | 1.45 (1.08,1.93) |
| 20-24 | 1.04 (0.88,1.24) | 0.97 (0.79,1.18) |
| 25-34 | *1.00 (Reference)* | *1.00 (Reference)* |
| 35-39^c^ | 1.26 (1.06,1.49) | 1.30 (1.09,1.55) |
| ≥40^c^ | 1.61 (1.21,2.15) |  |

^a^Infants with completely recorded infant and maternal characteristicsc; ^b^Infants with completely recorded infant, maternal and birth characteristics; ^c^A single risk ratio for both maternal age groups 35-39 and ≥40 years was calculated in sensitivity analyses due to low numbers. Abbreviations: CDH, congenital diaphragmatic hernia; CI, confidence interval.


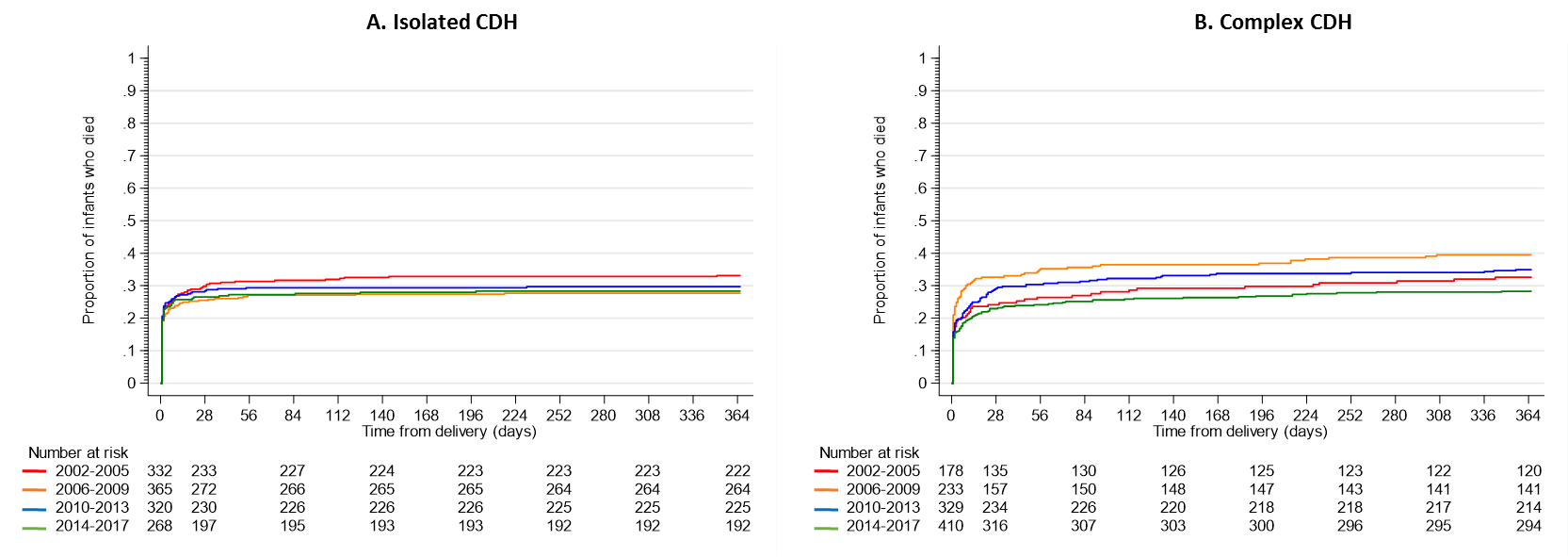

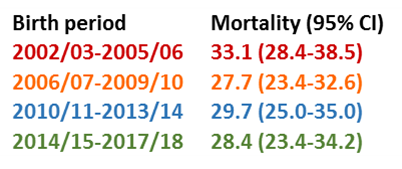

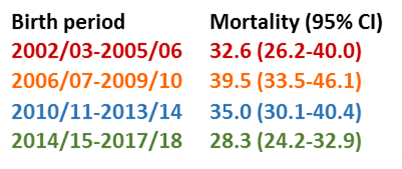


**Supplementary Figure 1 – One-year mortality estimates by CDH subtype and birth period. (A)** Isolated CDH **(B)** Complex CDH. Abbreviations: CDH, Congenital diaphragmatic hernia; 95% CI, 95% confidence interval.


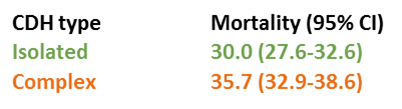


**Supplementary Figure 2 – Ten-year mortality estimates by CDH subtype.** Abbreviations: CDH, Congenital diaphragmatic hernia; 95% CI, 95% confidence interval.
